# The Genomic Complexity of a Large Inversion in Great Tits

**DOI:** 10.1093/gbe/evz106

**Published:** 2019-05-22

**Authors:** Vinicius H da Silva, Veronika N Laine, Mirte Bosse, Lewis G Spurgin, Martijn F L Derks, Kees van Oers, Bert Dibbits, Jon Slate, Richard P M A Crooijmans, Marcel E Visser, Martien A M Groenen

**Affiliations:** 1 Animal Breeding and Genomics, Wageningen University & Research, Wageningen, The Netherlands; 2 Department of Animal Ecology, Netherlands Institute of Ecology (NIOO-KNAW), Wageningen, The Netherlands; 3 Swedish University of Agricultural Sciences (SLU), Uppsala, Sweden; 4 Department of Molecular and Cellular Biology, Harvard University; 5 School of Biological Sciences, University of East Anglia, Norwich Research Park University of East Anglia, Norwich, United Kingdom; 6 Department of Animal and Plant Sciences, The University of Sheffield, Sheffield, United Kingdom

**Keywords:** songbird, structural variation, CNVs, *Parus major*

## Abstract

Chromosome inversions have clear effects on genome evolution and have been associated with speciation, adaptation, and the evolution of the sex chromosomes. In birds, these inversions may play an important role in hybridization of species and disassortative mating. We identified a large (≈64 Mb) inversion polymorphism in the great tit (*Parus major*) that encompasses almost 1,000 genes and more than 90% of Chromosome 1A. The inversion occurs at a low frequency in a set of over 2,300 genotyped great tits in the Netherlands with only 5% of the birds being heterozygous for the inversion. In an additional analysis of 29 resequenced birds from across Europe, we found two heterozygotes. The likely inversion breakpoints show considerable genomic complexity, including multiple copy number variable segments. We identified different haplotypes for the inversion, which differ in the degree of recombination in the center of the chromosome. Overall, this remarkable genetic variant is widespread among distinct great tit populations and future studies of the inversion haplotype, including how it affects the fitness of carriers, may help to understand the mechanisms that maintain it.

## Introduction

Inversions are structural intrachromosomal mutations resulting in the reversal of gene/sequence order. Chromosomal inversions represent an important class of polymorphism that are of particular interest in evolutionary studies (Hoffmann and Rieseberg 2008; Kirkpatrick 2010). Numerous studies have shown inversions to be important factors in speciation and adaptation (reviewed in Hoffmann and Rieseberg 2008). Studies of hominin evolution indicate a role of inversions in the process, with more than 1,000 inversions arising in both the human and chimpanzee lineages because they shared a common ancestor (Hellen 2015). Red fire ants (*Solenopsis invicta*) provide an interesting example of how inversions can promote adaptation; whether or not ant colonies contain a single queen or multiple queens depends on which inversion genotype is present the colony. The two social forms are genetically isolated (Keller and Ross 1998; Wang et al. 2013). In passerines, inversions are significantly more common in clades with more sympatric species, which suggests that inversions may often evolve or be maintained because they suppress recombination between the genomes of hybridizing species (Hooper and Price 2017). In both the white-throated sparrow (*Zonotrichia albicollis*) and the ruff (*Calidris pugnax*), morphs with different sexual behaviors are determined by inversions (Küpper et al. 2016; Lamichhaney et al. 2016; Tuttle et al. 2016). The inversion in the white-throated sparrow is very large, harboring ≈1,000 genes, and lethal in homozygous state (Tuttle et al. 2016).

To explain how inversions are maintained in a population it is important to understand the different mechanisms underlying selection on inversions. There can be meiotic drive if the inversion harbors alleles that alter segregation distortion (Kirkpatrick and Barton 2006). Selective advantages can also occur when an inversion affects the expression of advantageous genes located within or closely linked to the inversion (Puig et al. 2004). The effect of the inversion on gene expression is well-documented in red fire ants (Wang et al. 2008, 2013; Nipitwattanaphon et al. 2013; Lucas et al. 2015; Huang et al. 2018). In this species, gene expression differences between the monogyne and polygyne social forms are greatest in the inversion, suggesting that the inversion plays a key role in morphological and behavioral differences between the two forms. In addition, selective advantages of an inversion can be the result of recombination disruption in heterozygotes, which can preserve advantageous alleles. Moreover, reduced crossing-over within the inversion is associated with higher recombination rate elsewhere in the genome (Stevison et al. 2011), which in turn can modulate selection (McGaugh et al. 2012).

In many cases, recombination is suppressed between an inverted haplotype and the wild haplotype (Butlin 2005; Kirkpatrick and Barton 2006; Hoffmann and Rieseberg 2008; Kirkpatrick 2010). As a result of this lack of recombination in heterozygous inversion carriers, strong linkage disequilibrium (LD) between loci within the inverted region can rapidly build up. Although the lack of recombination can maintain advantageous variants without disruption throughout generations (i.e. supergenes, reviewed in Thompson and Jiggins 2014), there are also possible costs associated with the suppression of recombination. Each of the inversion haplotypes will behave as a single heritable entity that can help to retain certain alleles in the population even when they are subject to purifying selection (i.e. deleterious recessive alleles can be maintained if they are found within inversion polymorphisms by a ‘hitchhiking’ effect, Kirkpatrick and Barton 2006). As a consequence, deleterious recessive alleles can accumulate in regions of low recombination, such as an inversion, as they are no longer effectively removed by purifying selection. Moreover, throughout evolution an inversion becomes structurally more complex than the noninverted counterpart and often experiences a degenerative process (Tuttle et al. 2016). This degenerative process has been reported to be associated with a size increase in young supergenes (Stolle et al. 2018). In general, an increase in the number of gene copies can alter *trans*- and *cis*- gene expression, which might generate novel phenotypic variation (Geistlinger et al. 2018).

Inversions may harbor complex genomic rearrangements at their breakpoints (Calvete et al. 2012), given that inversion breakpoints are more likely to happen at complex parts of a chromosome (Carvalho and Lupski 2016). Apart from changing the gene order, inversions also often involve gene duplications that can lead to genetic novelty and subsequent adaptation (Furuta et al. 2011). In mosquitoes from the species complex *Anopheles gambiae*, haplotypes involving structural rearrangements at the breakpoint of a paracentric inversion have shed light on the origin and evolution of their malaria vectorial capacity (Sharakhov et al. 2006). The presence of repetitive regions at inversion breakpoints is recurrent and in fact both inversions and repetitive regions can share the same mechanism of formation, such as non-allelic homologous recombination (NAHR; Kehrer-Sawatzki and Cooper 2008; Carvalho and Lupski 2016). Understanding structural variations linked to inversion breakpoints may help to clarify the possible functionality and evolutionary history of inversions.

Genetic markers like SNPs and sequence data can be used to identify inversions polymorphism given the distinct population genetic structure caused by LD patterns within inversions. Thus, methods that are based on principal components analysis (PCA) can detect the unusual genetic structure of inversions (Ma and Amos 2012). In this study, we describe a 64.2 Mb putative inversion on Chromosome 1A in great tits (*Parus major*), a widely studied songbird in ecology and evolution (Visser et al. 1998; Kvist et al. 2003; Husby et al. 2011) with a broad range of genomic resources such as a high density SNP array (Kim et al. 2018), reference genome and methylome analysis (Laine et al. 2016) as well as copy number variation (CNV) maps (da Silva et al. 2018; Kim et al. 2018).

## Materials and Methods

### Population Description, Genotyping, and Sequencing

A total of 2,322 great tits were genotyped using a custom made Affymetrix great tit 650 K SNP chip (Kim et al. 2018) at Edinburgh Genomics (Edinburgh, United Kingdom). SNP calling was done following the Affymetrix best practices workflow by using the Axiom Analysis Suite 1.1. After sample filtering, 26 birds with dish quality control (Nicolazzi et al. 2014) <0.82 and SNP call rate <95% were discarded. SNPs with minor allele frequency (MAF) <1% and call rate <95% were removed. Only autosomes were used in this study. After filtering, 2,296 birds and 514,799 SNPs were kept for subsequent analysis. The genotyped birds were from our long-term study populations on the “Veluwe” area near Arnhem, the Netherlands (52^∘^02ʹN, 5^∘^50ʹE). More information regarding the origin of the birds and the in vitro DNA procedures are described by da Silva et al. (da Silva et al. 2018). The raw genotype data used in this study were submitted to GEO (GSE105131). Filtered genotypes and the source code to perform all analyses described below are available at Open Science Framework (https://osf.io/t6gnd/? view_only=821507ec135b44778d8b80254c24633b; last accessed 5 June 2019).

In addition to the birds genotyped on the SNP chip, we also used sequence data from 29 birds (10 from the Wytham Woods population in Oxford [UK], 19 birds sampled from 15 other European populations). Each bird was sequenced at an average depth of around 10× using paired-end sequencing libraries. Details of sequencing analysis, as well as information regarding the origin and sample quality of each bird are provided elsewhere (Laine et al. 2016).

### Identification and Characterization of a Large Inversion on Chromosome 1A

Population structure between SNP-typed individuals was explored using a PCA approach, previously applied for the study of inversions (Ma and Amos 2012), using the snpgdsPCA function in SNPRelate R/Bioconductor package (v. 1.10.2) (Patterson et al. 2006; Zheng et al. 2012). Each autosome was analyzed separately.

Following PCA, we estimated the fixation index (*F_ST_*) in a SNP-wise fashion, using the Fst function available in snpStats R/Bioconductor package (v. 1.26.0) (Clayton 2015) to compare birds in different clusters identified by visual inspection (i.e. subpopulations) of PCA plots. As SNP heterozygosity is expected to be higher within the inversion in carriers (i.e. birds with two different inversion haplotypes), the ratio of heterozygous birds (i.e. “AB”) for each SNP was assigned within each subpopulation. The SNP-wise *F_ST_* and heterozygosity values were used to define the likely breakpoints of the inversion.

Pairwise D′ values (Lewontin and Kojima 1960), using all birds, were calculated to assess LD patterns on Chromosome 1A. To aid visualization of the patterns revealed by the SNP data, SNPs were pruned to retain loci with MAF >0.4 and an LD threshold of 0.05 (using genomic windows with a maximum size of 500 kb). Pruning was performed with the snpgdsLDpruning and snpgdsLDMat functions within the SNPRelate R/Bioconductor package (v. 1.10.2) (Zheng et al. 2012). A total of 214 SNPs was retained and used in the LD analysis plot. We produced a graphical representation of the LD map using the LDheatmap function from the LDheatmap R package (v. 0.99-2; Shin et al. 2006). The function used to infer LD in this study makes use of the expectation-maximization (EM) algorithm (Excoffier and Slatkin 1995), which is able to infer LD from unphased data. In addition, the *R*^2^ (Zaykin et al. 2008) estimator was used for comparison with results from D′ because each estimator may respond differently to low-frequency alleles (Wray 2005).

### Inference of Structural Complexity at Chromosome 1A

We used CNV data obtained from SNP intensity information from the same great tit population in the Netherlands, as described previously (da Silva et al. 2018), to evaluate if certain CNVs are associated with normal/inverted phases. Moreover, we identified CNVs in the 29 resequenced birds from different European populations (Laine et al. 2016). First, we used the .*bam* file of each sample, containing reads mapped onto the reference genome build 1.1 using BWA (Li and Durbin 2009), to extract map locations with samtools (Li et al. 2009) as described in CNV-seq manual (Xie and Tammi 2009). CNVs were called with the default parameters of CNV-seq (Xie and Tammi 2009). CNV-seq uses coverage information to calculate a ${\;\text{log}\;}_{2}$ transformed ratio between the subject samples (inv-norm only, because inv-inv birds were absent from the data set) and wild-type samples (norm-norm). A positive ratio is associated with copy number gain (duplication), whereas a negative ratio is associated with copy number loss (deletion).

In addition, we used Lumpy (Layer et al. 2014) with default parameters, incorporated in the speedseq pipeline (Chiang et al. 2015) to predict the exact breakpoints of the CNV events and to predict inversion events from sequence data. Information from split and discordant mapped reads was used to describe the structure of a CNV complex in one of the inversion breakpoints (details in the supplementary section “Patterns in Split Reads Supporting the CNV Complex,” Supplementary Material online).

### Inversion Detection by PCR-RFLP

As genotyping with SNP arrays can be time consuming and expensive, we designed an alternative method to type the Chromosome 1A inversion, based on a PCR followed by a restriction enzyme digestion (PCR-RFLP). For this, we used the SNP with the second highest *F_ST_* value (i.e. AX-100689781) because it almost perfectly captures the inversion (99.32% of the inv-norm birds have AB genotype and 98.95% of the norm-norm birds have the AA genotype). The SNP with the highest *F_ST_* value did not allow distinguishable fingerprints in silico because there are no restriction enzymes which differentially cut the two alleles. Instead, we choose SNP AX-100689781 which is located close to the downstream breakpoint of the inversion, at position 65,878,384 in the great tit genome build 1.1 (Laine et al. 2016; details in the supplementary section “Primer Design and Enzyme Search,” Supplementary Material online). This SNP is located within the first intron of the gene *PIK3C2G*. We genotyped 42 birds by PCR-RFLP which had also been genotyped with the SNP chip.

For each PCR-RFLP reaction, we used 6 μl of DNA (10 ng/μl). The PCR was performed with OneTaq 2X mastermix (New England Biolabs) and 1 μl of primermix (primer sequences are given in the supplementary section “Primer Design and Enzyme Search,” Supplementary Material online). The PCR program had steps of: 95 °C for 5 min, 34 cycles of 95 °C for 30 s, 55 °C for 45 s, 72 °C for 90 s and a final elongation step of 72 °C for 10 min. The digestion reaction was done for 5 h at 37 °C using 3 μl of the PCR product, 0.4μl of the enzyme *SspI* (10 U/μl, New England Biolabs), 1 μl of the *SspI* buffer 10X and 5.6μl of sterile deionized water (MQ). The PCR-RFLP was analyzed on a 3% agarose gel. The restriction fragments were checked on the Geldoc XR+(Biorad) gel documentation system with the software Image Lab (v. 5.2.1).

## Results

### Population Structure for Chromosome 1A Reveals a Large Inversion

**Fig. 1.**
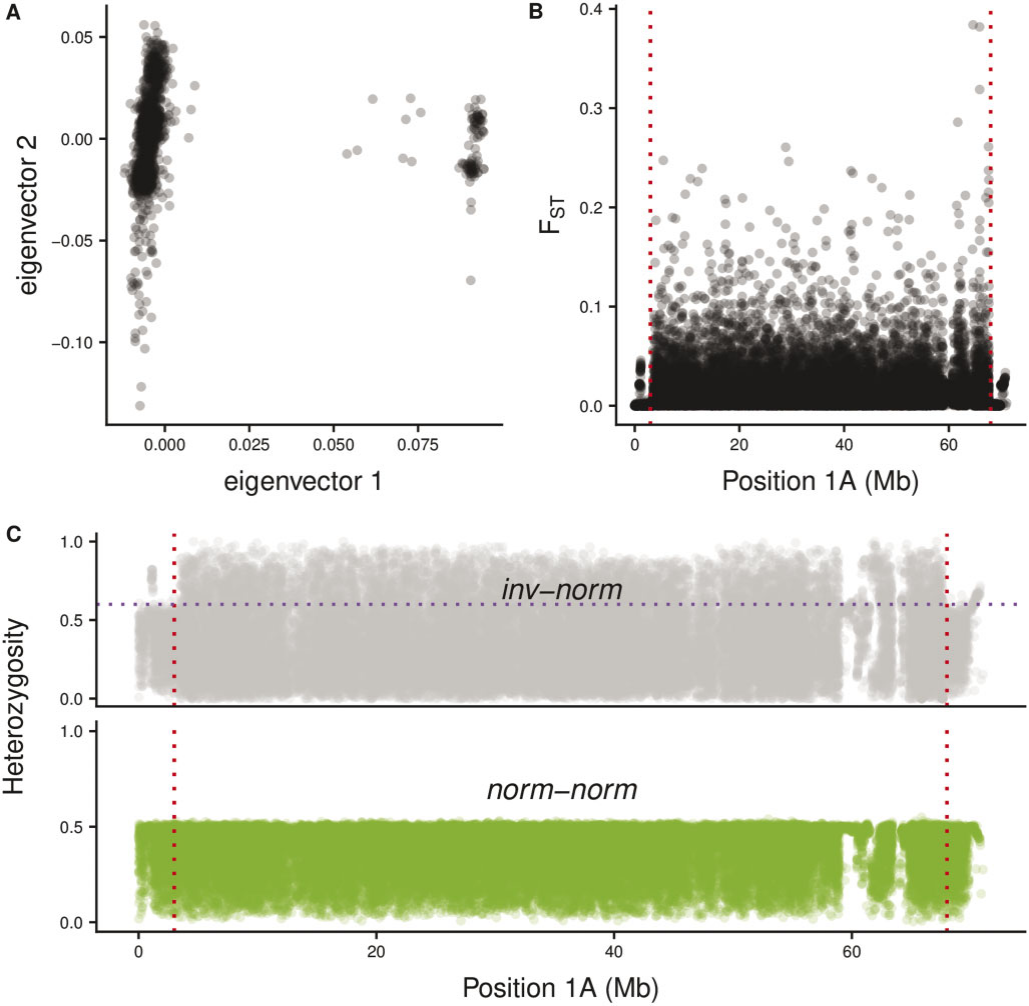
—(*A*) PCA: based on the SNPs located on Chromosome 1A, a principal component analysis revealed two distinct subpopulations. The distinction is given by Eigenvector One, which gave the initial evidence of inversion carriers. (*B*) *F_ST_*: these two subpopulations display highly differentiated SNPs across the whole of Chromosome 1A, except at regions near to telomeres. (*C*) Heterozygosity: each subpopulation exhibits a particular heterozygosity level across the Chromosome 1A. The inv-norm subpopulation has many SNPs with high heterozygosity within the region bounded by the tentative breakpoints given by *F_ST_* analysis (≈3–68 Mb, delimited by the red-dashed lines). The purple dashed line represents the maximum expected in norm-norm birds. SNPs above this threshold are considered informative.

We found a large putative inversion on Chromosome 1A. Based on visual inspection of the PCA (Patterson et al. 2006), we classified the clustering patterns separately for each autosome in the great tit genome (supplementary fig. 1, Supplementary Material online). Plots for whole chromosomes may reveal obvious substructure if the inversion is relatively large. Although additional chromosomes display some population structure (e.g. chromosomes 5 and 7, supplementary figs. S1 and S2, Supplementary Material online), the variation within PCA clusters is greater, and the *F_ST_* values across these chromosomes less conclusive, relative to the patterns seen on Chromosome 1A. Moreover, this unusual PCA pattern, which was most likely reflecting an inversion, was briefly reported elsewhere (Bosse et al. 2017). Therefore, the remainder of this article considers the likely inversion polymorphism on Chromosome 1A. Chromosome 1A displayed clear population structure for the first eigenvector (fig. 1*a*, First and Second eigenvectors explain 2.28% and 0.50% of the variance, respectively), with two subpopulations that are genetically distinct. The larger subpopulation comprises 2,179 birds and the smaller one contains only 117. Among these 117 birds, 10 display intermediate values in Eigenvector One. Analysis of the genotypes of these 10 birds indicates that they are carrying a distinct copy of the inversion that is derived, possibly by gene conversion, from the most common inversion haplotype (i.e. the 10 being heterozygotes and the remainder being homozygous for the inversion haplotype). The genotypes and LD patterns in the center of the inversion are discussed in detail in a subsequent section (i.e. LD and haplotypes across the inversion).

We obtained high *F_ST_* values between the two PCA plot subpopulations across almost the whole of Chromosome 1A except for the most distal SNPs on the chromosome (fig. 1*b*). The heterozygosity level in each of these subpopulations across Chromosome 1A is also strikingly different (fig. 1*c*). The heterozygosity level for the smaller subpopulation is greater than for the larger subpopulation, except for markers close to the telomeres. This suggests that the smaller subpopulation contains birds heterozygous for the inversion polymorphism. The heterozygosity patterns are consistent with the pattern shown by the *F_ST_* analysis, in terms of where the inversion is located on the chromosome. In addition, the *F_ST_* values of the SNPs located on Chromosome 1A have a significantly different distribution than SNPs in the rest of the genome (Wilcoxon rank sum test with continuity correction *P* value ≈ 0.0002).

The PCA, *F_ST_*, and heterozygosity results support the existence of a pericentric inversion in the smaller PCA subpopulation (117 birds). This putative inversion comprises ≈90% of the length of the chromosome (≈64.2 Mb) and is present only in heterozygous state in this great tit population (given the PCA clustering in addition to the high levels of heterozygosity of the SNPs at Chromosome 1A in inv-norm birds, fig. 1*a–c*).

### LD and Haplotypes across the Inversion

**Fig. 2.**
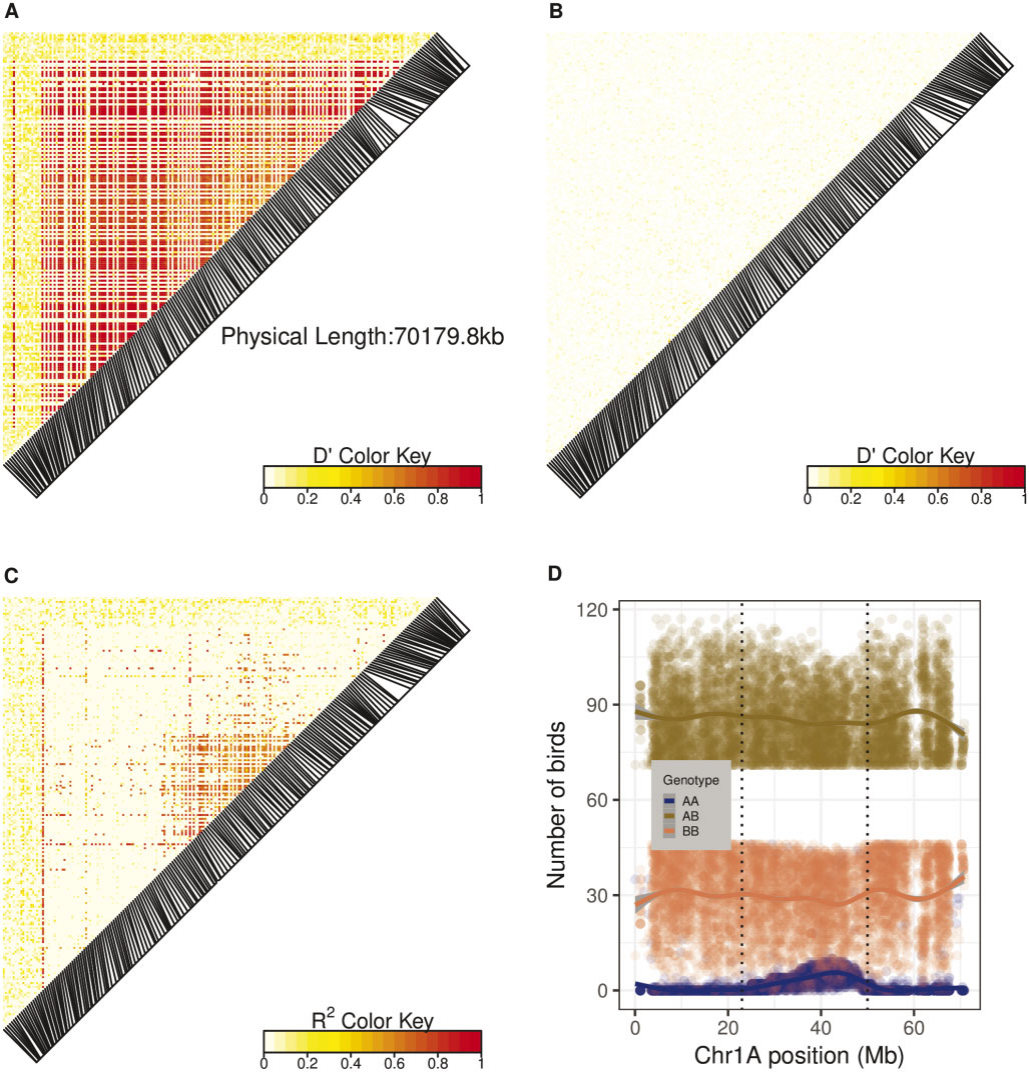
—The pairwise LD on the Chromosome 1A. (*A*) D′ measured in 2,296 great tits. (*B*) D′ measured in 2,179 norm-norm birds. Figures in the lower panels (*C* and *D*) support possible recombination events in the center of the inversion. In other words, possible recombination in the center of the inversion is supported by the distinct genotype distribution in comparison with the rest of the inversion and confirmed by *R*^2^. As *R*^2^ metric has reduced power to detect LD among SNPs with low allele frequency, the LD is reflected only in the center of the inversion. (*C*) *R*^2^ measured in 2,296 great tits reveals an LD block only in the middle of the chromosome. The full inversion does not show elevated LD, due to the limitation of *R*^2^ at dealing with low-frequency SNP alleles outside the center of the inversion. (*D*) Genotype frequency of informative SNPs (heterozygosity >0.6) across Chromosome 1A in the inv-norm subpopulation. The vertical dotted line roughly indicates the genomic region of middle block which harbors a higher number of birds with “AA” genotypes when compared with the rest of the inversion. Along with the LD pattern from *R*^2^ method, the genotype frequencies suggest a different genetic structure at the center of the inversion.

We used the unphased SNP genotypes from all birds to characterize LD across Chromosome 1A by calculating D′ (Lewontin 1964). As expected for regions with low recombination, a large LD block which overlaps the whole inversion was identified (fig. 2*a*). This LD block is not present in norm-norm birds (fig. 2*b*), suggesting that recombination is only restricted in birds heterozygous for the inversion. On the other hand, when *R*^2^ is used as a measure of LD inference, an LD block is only observed in the middle of the chromosome (from position ≈24.6 to 48.8 Mb, fig. 2*c*). This *R*^2^ LD block overlaps the region that causes the two distinct genotype distributions among the 117 inv-norm birds (fig. 2*d*).

**Fig. 3.**
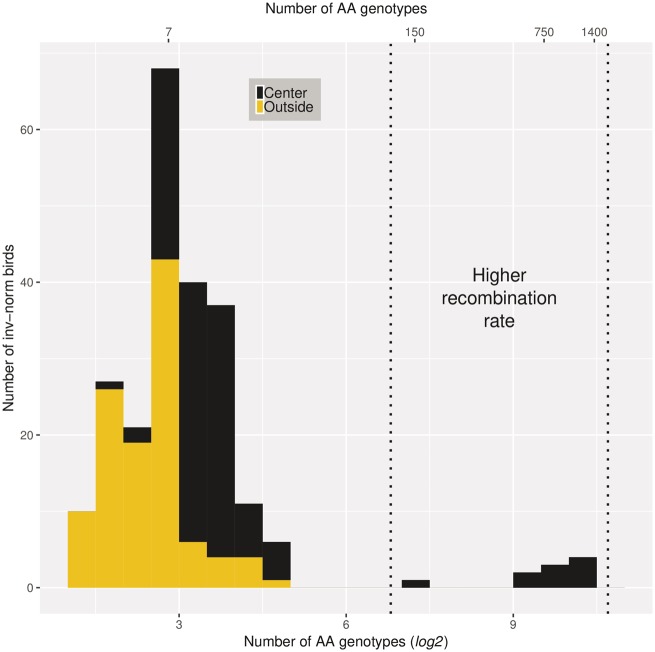
—Genotype distribution within/outside the center of the inversion (20–55 Mb) in inversion carriers. The number of genotypes is represented on a *log*_2_ scale to improve the visualization but untransformed values are shown on the upper *x* axis. Based on the number of “AA” genotypes it is possible to identify inv-norm-birds which harbor a different genotype distribution at the center of the inversion and therefore possibly have different inversion haplotypes (black bars among the dashed lines).

Initial results show that phasing procedures, such as BEAGLE, fail in inv-norm birds (data not shown). Consequently, these wrongly phased alleles could lead to wrong conclusions about inversion sequences. Therefore, a detailed analysis of genetic diversity within the different inversion haplotypes was not possible. Instead, we used genotype information to explore putative inversion haplotypes. In the center of the inversion (a 20–55 Mb window was used, which is a 5 Mb up- and downstream extension of the LD block in the center due to uncertainty over the precise breakpoint locations), the genotype frequencies (i.e. the ratio of genotypes “AA,” “AB,” and “BB,” where “A” is the major and “B” the minor allele in the general population) is substantially different between the ≈10% of the inv-norm birds (10 birds, supplementary fig. S5, Supplementary Material online) and the remainder of the inv-norm birds. The number of “AA” SNP genotypes (i.e. homozygous for the major allele, which is rare in the inversion) in these 10 inv-norm birds that differ from the others is greater than in the other inv-norm birds. A total of 107 birds (91.4%) have between 4 and 30 (mean = 11.61, standard deviation = 4.95) SNPs with genotype “AA” whereas the remaining 10 birds have substantially more “AA” genotypes (range = 146–1,382; mean = 892.4; standard deviation = 394.2; fig. 3). To a certain extent the 10 birds with distinct haplotypes can also be distinguished from the other inv-norm birds, by the PCA analysis due to their intermediate values in eigenvector one (0.053–0.076). These 10 birds are from four different areas in Netherlands (two birds from Buunderkamp; three birds from Westerheide; two birds from Roekelse Bos; two birds from Hoge Veluwe and one birds from an unknown location).

### Complex Genomic Structure at the Inversion Breakpoint

**Fig. 4.**
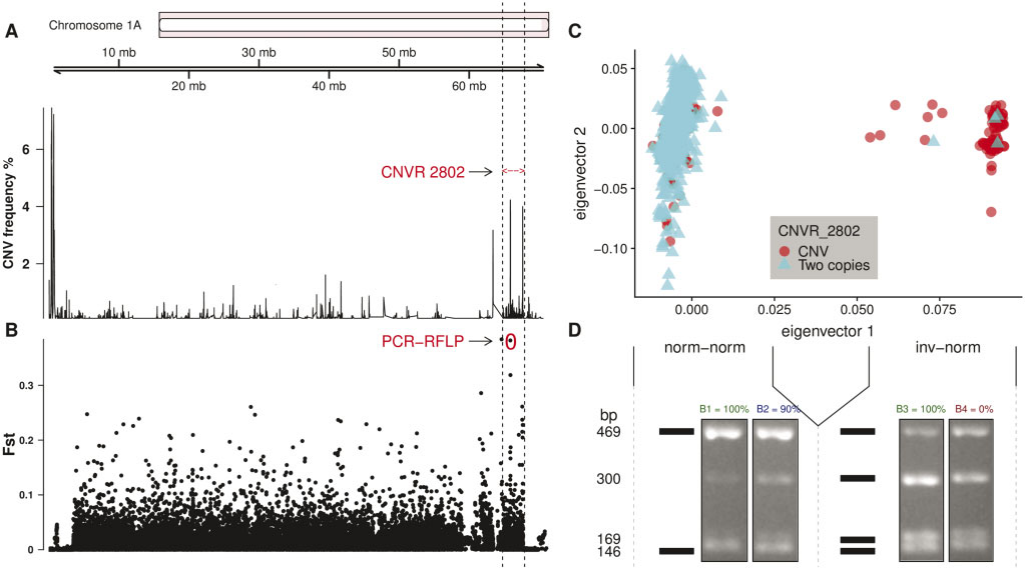
—CNVs in the inversion breakpoint. (*A*) CNV frequency across the Chromosome 1A and the genomic interval of the previously identified CNV region “2802” (≈64.83–67.67 Mb; da Silva et al. 2018), which is located at the inversion breakpoint. (*B*) *F_ST_* values across the chromosome. A red circle is highlighting the SNP used to the PCR-RFLP analysis. (*C*) A CNV in the inversion breakpoint is present in the vast majority of inv-norm birds whereas is rarely found in norm-norm birds. (*D*) Digestion pattern of the PCR-RFLP at the SNP AX-100689781. The black bars represent the expected gel patterns alongside each of the two observed patterns in each subpopulation (i.e. norm-norm and inv-norm). Distinct copy number genotypes are evidenced by the allele intensities in the gel after electrophoresis. The values above each gel picture depicts the fingerprint name and the degree of confidence to tag a specific karyotype state (i.e. percent of the birds with concordant inversion genotype between SNP array and PCR-RFLP). Green was used in highly confident profiles, blue in the medium confidence one, and red for B4, which has high heterozygosity (expected in inv-norm) but was only identified in two norm-norm birds. To differentiate between fingerprints note the distinct intensities of subsets of bands; between B1 and B2 the greatest difference is mainly at the 300/169 bp bands and between B3 and B4 the greatest difference is between the 469/300 bp bands.

Inversion breakpoints can provide insight in the evolutionary history of the inversion (Sharakhov et al. 2006). The downstream breakpoint of the Chromosome 1A inversion harbors a previously identified CNV region, “2802,” located at position 64.83–67.67 Mb (fig. 4*a*, da Silva et al. 2018). Of all 2,296 birds analyzed for the inversion, 2,021 were also previously analyzed for CNVs. This includes 1,921 birds classified as norm-norm and 100 as inv-norm. Among the norm-norm birds, 217 harbor CNVs at the downstream inversion breakpoint (11.29%) whereas 1,704 have two copies as expected in the diploid state. In contrast, 96% of the inv-norm birds have an individual CNV call mapped at the CNVR 2802. At this CNVR, 94.8% of all individual CNV calls are gains.

### Inversion Detection with PCR-RFLP

We looked for SNPs with the highest *F_ST_* possible, which concomitantly allowed different DNA fingerprints of their SNP genotypes to be obtained by restriction digest. For the SNP with the second highest *F_ST_* value (fig. 4*b*), “AA” and “AB” genotypes (i.e. associated with norm-norm and inv-norm karyotypes, respectively), our genotype assay produced two distinct in silico profiles when the PCR fragments were digested by the enzyme *SspI* (fig. 4*d*, represented by the black bars). The SNP is located in the first intron of the *PIK3C2G* gene. In a diploid region, we would expect a profile with four bands (i.e. “AB”) in an inv-norm bird whereas a profile with two bands (i.e. “AA”) would be norm-norm. However, as the SNP is placed in a repetitive region (i.e. containing a CNVR and segmental duplications), the obtained profiles are more complex. We obtained instead four different profiles, which differ in the intensity in each of the four possible fragments (fig. 4*d*). Profile B3 was only identified in inv-norm samples whereas the profiles B1, B2, and B4 were mostly, but not exclusively observed in norm-norm samples. However, birds with the profile B2, in 90% of the cases, are norm-norm and in 10% inv-norm. Unexpectedly, the profile B4, which shows high heterozygosity as in the inversion, was only identified in two norm-norm birds (0% of confidence, that is expected to be found in inv-norm but only found in norm-norm birds).

### Assessing Breakpoint Complexity from Sequencing Data

We classified 29 birds for the inversion from distinct European populations by whole genome resequencing (Laine et al. 2016) based on the presence of the CNV complex at the breakpoint. A total of 27 birds were classified as norm-norm and two as inv-norm. We used sequencing data from the two inv-norm birds, one from France and another from Belgium, to characterize CNVs across the inversion. At the downstream breakpoint, we detected a CNV (gain state) in both birds in agreement with the results from the Dutch great tit population, which suggests a high correlation of the inversion with a gain state at the downstream breakpoint (fig. 4*c*). None of the other 27 resequenced birds without the inversion showed CNVs at this region. The CNVs that we identified in the two inv-norm resequenced birds point to a substantial increase in the number of copies instead of only a single copy gain. The *log*_2_ values from CNV-seq at that region suggest around 10 copies in the inverted phase involving three CNVs that are part of the same structural complex (the regions among 65.87–65.90, 67.56–67.58, and 67.64–67.65 Mb, which together comprise ≈50.43 kb). In addition, we identified an increase of around 100 copies in a region upstream to the CNV complex (63.44–63.46 Mb, ≈20 kb), which in turn is followed by an increase of around 10 copies (63.46–63.56 Mb, ≈100 kb). It is unclear if these events are part of the same complex (supplementary fig. 4, Supplementary Material online shows the estimated number of copies in each of the abovementioned CNV regions). Considering only the three CNVs which are part of the complex, the inverted Chromosome 1A is at least 500 kb larger than the reference (i.e. the normal noninverted) haplotype. However, summing the CNV complex with other upstream CNV regions that are also only present in sequenced inv-norm birds (i.e. a region with ≈100 copies followed by other regions with ≈10 copies) suggests that the inverted chromosome may be up to 3.5 Mb larger than the normal chromosome.

**Fig. 5.**
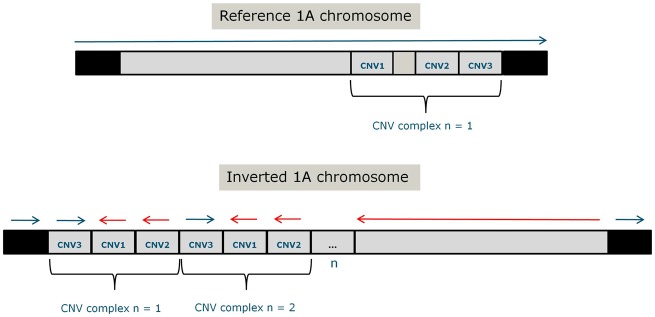
—Representation of the whole Chromosome 1A with the complex structural rearrangement in the downstream breakpoint of the inversion. Blocks in gray represent the inversion region whereas those in black are genomic regions outside the inversion. CNVs identified by sequencing in the two inv-norm birds which were sequenced are labeled as CNV1-3 for simplicity. Horizontal curly brackets define the structural complex which encompasses CNVs 1-3. The above chromosomal representation displays the chromosome as shown in the reference genome (Laine et al. 2016). The below representation displays the expected genomic structure in the inversion. CNVs are relatively larger than their real length for schematic purposes.

As split reads from sequencing data are useful to reveal complex rearrangements in the genome, we evaluated their pattern in the CNVR. We identified split reads in this region that support a complex genomic rearrangement involving different CNVs. Split reads and discordantly mapped paired reads show that this region contains a complex rearrangement of three intervals which are arranged in a different order and orientation when compared with the reference genome (supplementary section “Patterns in Split Reads Supporting the CNV Complex,” Supplementary Material online and fig. 5).

In addition, Lumpy (Layer et al. 2014) was used to predict the exact breakpoints of the inversion. We were unable to infer the whole inversion event from sequencing data, but interestingly one large inversion was unique to the two inv-norm samples that were sequenced. The inversion boundaries are from 62.15 to 63.55 Mb, with a length of 1.4 Mb on the reference genome. For the two inv-norm samples, nine (sample name = 233) and eight (sample name = 973) reads supported this 1.4 Mb inversion event. The coordinates of the inversion start lies within a single copy region, whereas the coordinates of the inversion end are located in the CNV complex (65.87–67.65 Mb). Therefore, we hypothesize that at least one of the inversion breakpoints is within the large complex; however, the precise coordinates are difficult to predict.

### Gene Content and Functionality at the Inversion Breakpoint

Genomic regions around the inversion breakpoints can have a different structure and nucleotide diversity compared with the rest of the inversion (Andolfatto et al. 2001; Hoffmann and Rieseberg 2008; Branca et al. 2011). The CNV complex overlaps 32 genes associated with a broad range of phenotypes in other species (for details on the phenotypes associated with each gene, see supplementary section “Genes Overlapping the CNVR at the CNV Complex,” Supplementary Material online). It is perhaps noteworthy that three genes (*BPGM*, *CALD1*, and *PIK3C2G*) could potentially be broken in the inverted haplotype, given that sequencing data shows CNVs only partially overlapping them.

## Discussion

Here, we have described a large putative inversion on Chromosome 1A of the great tit (Bosse et al. 2017) that covers more than 90% of the chromosome and contains almost 1,000 genes. The inversion is present in 5% of the analyzed Dutch population as well as in 2 out of 29 resequenced individuals from other European populations; one carrier was from Belgium and the other from France, indicating that the inversion is present in other great tit populations as well. In this study, the inversion was analyzed with a SNP array and by shotgun sequencing. Although the most likely explanation for suppressed recombination is an inversion (Kirkpatrick 2010), we acknowledge that methods such as FISH (Bishop 2010) and long read sequencing (Shao et al. 2018) need to be used to confirm the inversion hypothesis. It is feasible, though unlikely given the size of the region, that suppressed recombination leading to chromosomal divergence could arise without a chromosomal inversion (Bergero et al. 2007, 2008, 2013; Natri et al. 2013). For clarity in this discussion, we refer to the putative inversion found here simply as inversion.

In the population from the Netherlands, among the 2,296 birds analyzed after filtering, no homozygous bird for the inversion on Chromosome 1A was found. Given that very large inversions can cause homozygous lethality in songbirds (Tuttle et al. 2016), we investigated if this great tit population has significantly fewer homozygous inverted birds than expected. However, given the low frequency of the inversion, and assuming Hardy–Weinberg equilibrium (HWE), we would expect less than two homozygous inverted birds and it is thus unclear whether the complete absence of homozygotes is due to a deleterious recessive effect of the inversion or whether homozygotes are present in the population but not sampled in this study. A possible lethal effect of this inversion could be tested by exploring the frequency of genotypes among offspring of mated carriers. Given the structural complexity and large size of this inversion, a relevant biological effect could be expected. A CNV complex located at the downstream breakpoint encloses 32 genes involved in a wide range of biological processes, which could significantly change the amounts of the transcripts/proteins due to copy number changes in the genes located at the CNV complex. Future studies of this inversion polymorphism will be directed to test the lethality hypothesis and to measure the relative fitness of wild-type homozygotes, inversion carriers and inversion homozygotes. Indeed, this future goal was one motivation for developing a cheap and quick method (based on PCR-RFLP) to more easily type inversion karyotypes.

To identify the inversion without SNP array data, we selected the SNP with highest *F_ST_* value that concomitantly would produce a PCR-RFLP profile capable of distinguishing between inversion carriers and non-carries. The selected SNP is located at the first intron of the *PIK3C2G* gene, which is within the CNV complex at one of the putative inversion breakpoints. Along with *PIK3C2G*, several other genes are also located in the CNV complex and these genes have crucial roles in a broad range of processes from cell cycle to gene silencing (Supplementary section “Genes Overlapping the CNVR at the CNV Complex,” Supplementary Material online). Resequenced birds showed a high number of copies within that genomic region (≈10 copies in two inv-norm birds). Moreover, the PCR-RFLP gel intensities support at least four genotypes (three for norm-norm and one for inv-norm birds). Thus, this substantial copy number change in inv-norm birds could underlie distinct patterns in gene expression and consequently phenotypic variation. Interestingly, such complex rearrangements at inversion breakpoints have key evolutionary roles in other species, for example an effect on malaria vectorial capacity in mosquitoes (Sharakhov et al. 2006).

A CNV complex located at the breakpoint seems to be older than the inversion. Assuming a single origin for this complex, the CNV sequences may be older than the inversion given that it is present in virtually all inv-norm birds whereas it occurs at low frequency in norm-norm birds. More than 10% of the norm-norm birds have at least one CNV overlapping the CNV complex. In addition, a repetitive structure is usually found at inversion breakpoints underlying their mechanisms of formation (such as NAHR; Hoffmann and Rieseberg 2008; Carvalho and Lupski 2016). Thus, it is possible that the inversion is a result of the CNV sequences, which underpinned the mechanism of the inversion formation. However, it remains possible that CNVs are present in the inversion only due to a “hitchhiking” effect and thus did not necessarily contribute to the inversion’s formation. The hypothesis that CNVs might have underpinned the formation of the inversion remains speculative and needs further investigation. Considering the size of all CNVs associated with the inversion (i.e. complex with ≈10 copies and another complex of ≈10 copies with an additional region with ≈100 copies, identified by sequencing) the inverted chromosome is estimated to be ∼3.5 Mb larger than the reference sequence reported in genome build 1.1. The greater length of chromosomes harboring the inversion is in line with the hypothesis of degenerative expansion in young supergenes (Stolle et al. 2018). However, genetic variation is not only present in the CNV complex but also at the center of the inversion.

Allele phasing in inv-norm birds is challenging because phasing strategies like BEAGLE assume HWE Browning and Browning (2007); this assumption is often violated at inversion genotype-informative SNPs (i.e. the vast majority of the genotype-informative SNPs significantly deviate from HWE). Thus, we used the genotype distribution (i.e. the proportions of “AA,” “AB,” and “BB,” genotypes) to partially explore the haplotypes in the inversion. There are at least two (and perhaps three or more) putative inversion haplotypes, which are reflected by the number of AA genotypes at the center of the inversion (located at ≈20–55 Mb of the Chromosome 1A, fig. 3, note the log scale and three distinct groups). In the LD analysis, only the *R*^2^ metric reflected the variation within inv-norm birds. This variation derives from the SNPs that are located in the center of the inversion (i.e. LD block in the center, fig. 2*c* and *d*). The *R*^2^ method has a constraint to deal with low-frequency alleles (Wray 2005) whereas D′ is not highly dependent upon allelic frequencies (Hedrick 1987). Interestingly, in the inv-norm population, the frequency of the less common genotype in the informative SNPs at the *R*^2^ LD block (fig. 2*a*) is not as low as in the rest of the inversion (fig. 2*b*). Thus, the distribution of allele frequencies in the inv-norm birds may explain why the *R*^2^ metric does not describe elevated LD, outside the center of the inversion, and is consistent with the hypothesis of a higher recombination rate in the center. In other words, because the two different LD measures are not equally sensitive to rare alleles, and because the allele frequencies seem to be different in the center of the inversion than elsewhere, one metric finds a pattern that the other misses. Presumably this is because occasional recombination has caused allele frequencies and LD patterns to be slightly different in the center than in the rest of the inversion. Due to the expected very low rates of recombination within the inversion in heterozygotes (Kirkpatrick 2010), we did not expect multiple haplotypes for the inversion. However, on timescales of 10^5^ generations or longer, even this limited recombination works as an important source of variation within inversions (Kirkpatrick 2010). Indeed, gene conversion and multiple crossing overs, at least far from the breakpoints, are possible within inversions (Andolfatto et al. 2001; Hoffmann and Rieseberg 2008; Korunes and Noor 2018). Thus, rare recombination events may explain distinct haplotypes found in the center of the inversion. Moreover, as CNVs can underlie mechanisms of formation and be prone to errors, independent inversion events and errors during meiosis cannot be discarded.

It is unclear whether the inversion has any phenotypic effects. Nevertheless, the CNVs identified by sequencing at the CNV complex directly overlap at least three genes, including *CALD1* involved in smooth muscle contraction (Walsh 1994), *BPGM* underlying oxygen sensing in blood cells (Petousi et al. 2014) and the abovementioned *PIK3C2G* gene (the other 29 genes overlap a CNVR in the same region but do not overlap partially CNVs identified by sequencing). On other songbird species, such as the zebra finch (*Taeniopygia guttata*), sperm morphology and motility is associated with an inversion in the Z Chromosome (Kim et al. 2017). Moreover, inversions in zebra finches can have strong additive effects on several morphological traits and increase mortality rates (Knief et al. 2016). In white-throated sparrows, which display different plumage morphs and sexual behavior, a large inversion involving up to 1,000 genes and lethal in its homozygous state, has a profound role in disassortative mating (Tuttle et al. 2016). However, there is no evidence of distinct morphs in great tit. Thus, if the inversion is underlying any kind of mate choice it may be reflected by a more subtle trait or behavior. Apart from songbirds, large inversions can underlie a number of phenotypes in nature, ranging from mimicry and crypsis in butterflies and moths (Nadeau et al. 2016) to meiotic drive in mice (Lyon 2003). Our detailed characterization of the variability and complexity of this large inversion provides the foundation for further studies aiming to discover the phenotypic effects and the evolutionary role of this inversion.

## Ethical Approval

This work was carried out under a license of the Animal Experimental Committee of the Royal Dutch Academy of Sciences (KNAW) protocol NIOO-10.07.

## Supplementary Material

Supplementary data are available at *Genome Biology and Evolution* online.

## Supplementary Material

Supplementary_Material_evz106

## Notes

**Data deposition:** The raw genotype data sets used during the current study are available at NCBI (https://www.ncbi.nlm.nih.gov/geo/query/acc.cgi? acc=GSE105131).
