# Supplementary material for: The Genomic Complexity of a Large Inversion in Great Tits
Source: Genome Biol Evol. 2019 May 22;11(7):1870–81. doi: 10.1093/gbe/evz106 (PMC6609730; doi:10.1093/gbe/evz106)
Supplement: Supplementary_Material_evz106 [file supplementary_material_evz106.pdf]

Contents

1 Supplemental figures **1**

2 Supplementary methods **5**
2.1 Classification confirmation for inversion carriers . . . 5
2.2 Selection of the SNP used in the RFLP-PCR . . . 5
2.3 Primer design and enzyme search . . . 5

3 Supplementary results **7**
3.1 Identification of the inversion carriers . . . 7
3.2 Quality of the SNPs used in the LD analysis . . . 7
3.3 Genes overlapping the CNVR at the CNV complex . . . 7
3.4 Patterns in split reads supporting the CNV complex . . . 9

List of Figures

1 **PCA for all autosomes** . . . 1
2 **Fst values in chromosomes 5 and 7** . . . 2
3 **Verification of inversion karyotypes** . . . 3
4 **CNV-seq at the inversion breakpoint** . . . 4
5 **Heatmap comparing inv-norm and norm-norm birds** . . . 4
6 **Expected amplicon cut patterns with SspI** . . . 6
7 **Split reads supporting the CNV complex** . . . 9

List of Tables

1 Primers used in the PCR-RFLP analysis. . . . 5
2 Genes overlapping the CNV complex at the downstream breakpoint of the inversion. . . . 8
3 Sequencing coverage in two inv-norm birds . . . 9

1 Supplemental figures

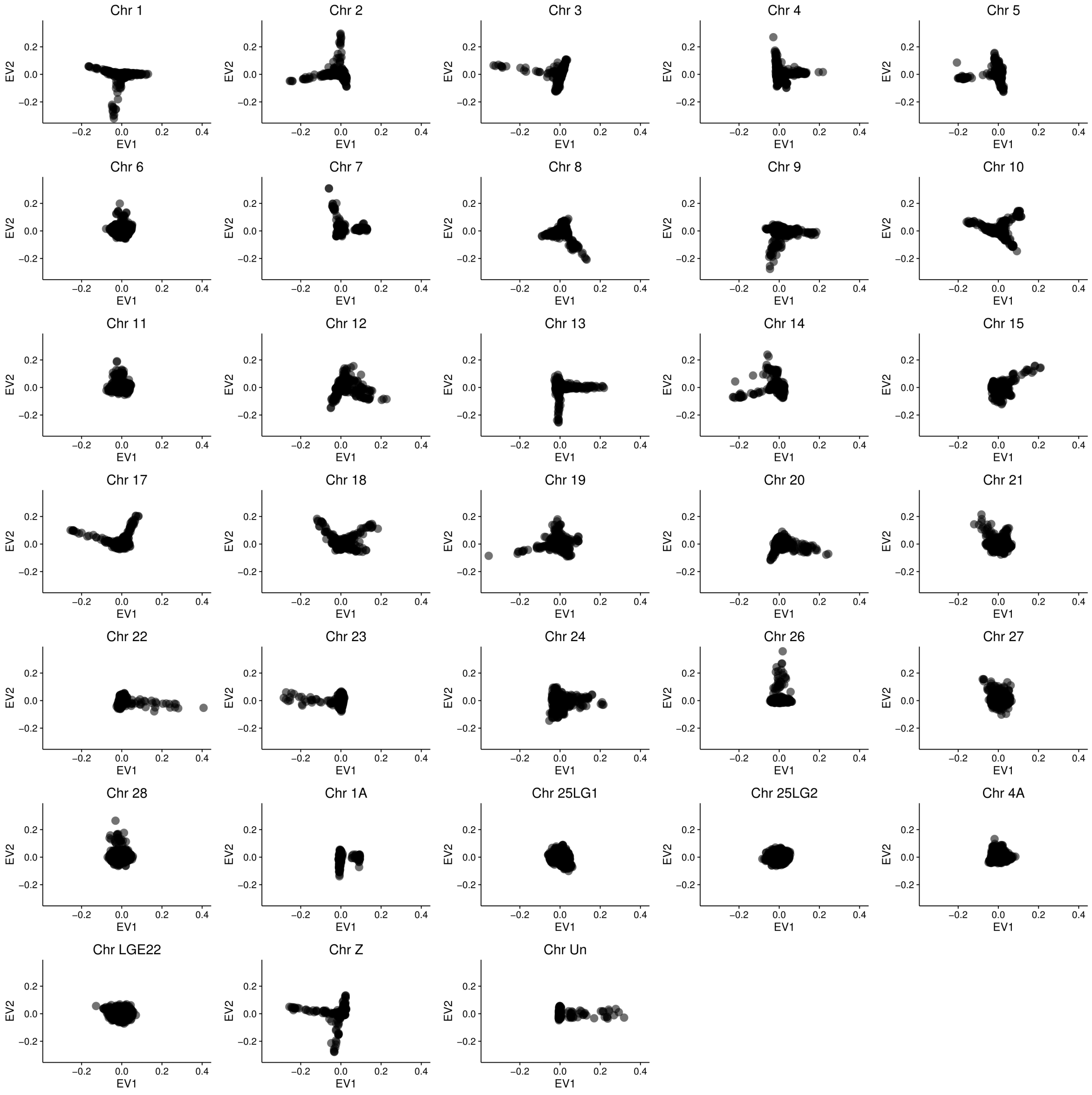

Figure 1: PCA for all autosomes in the great tit genome build 1.1.

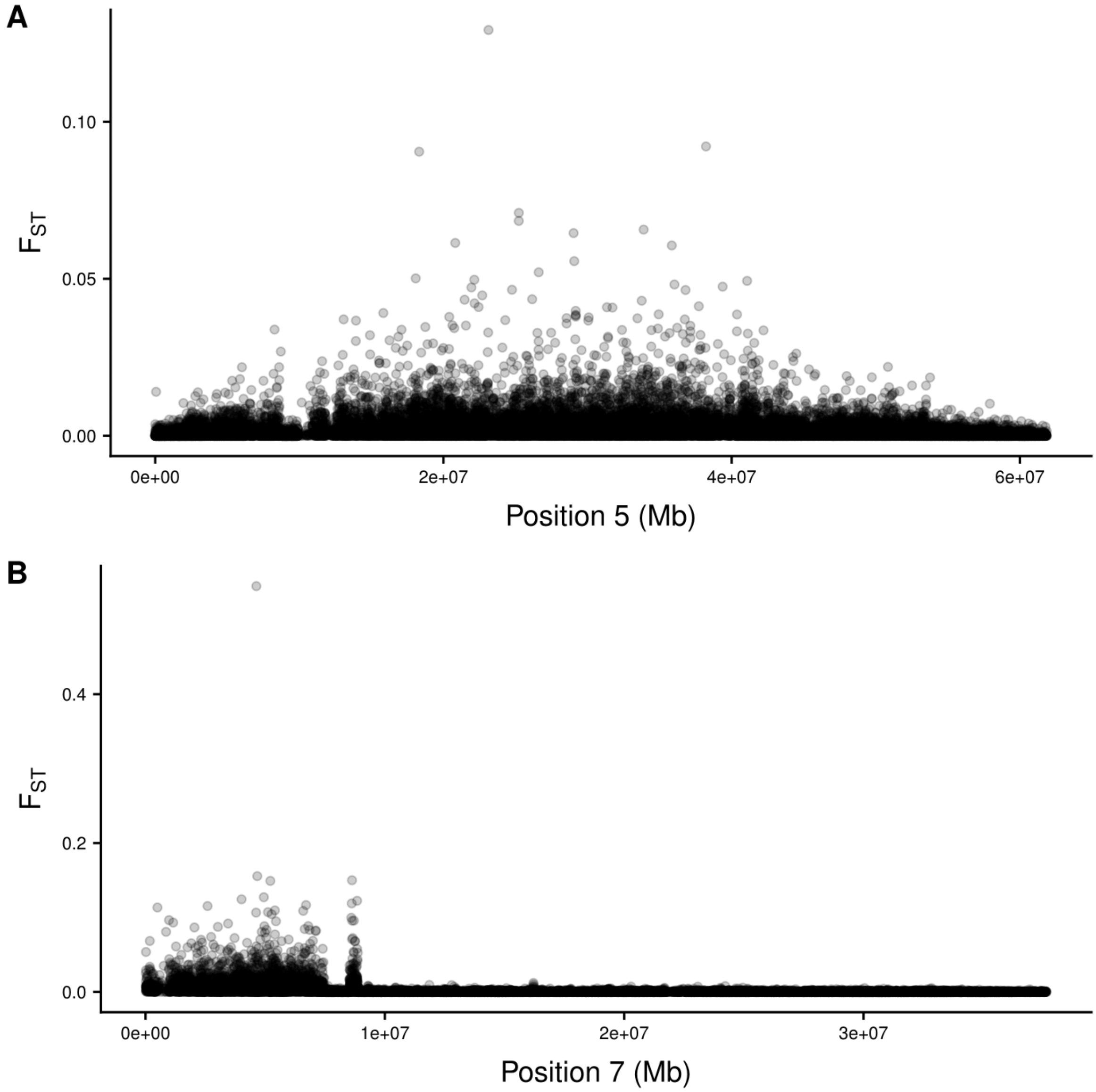

Figure 2: A-) F_{ST} across the Chromosome 5. B-) F_{ST} across the chromosome 7.

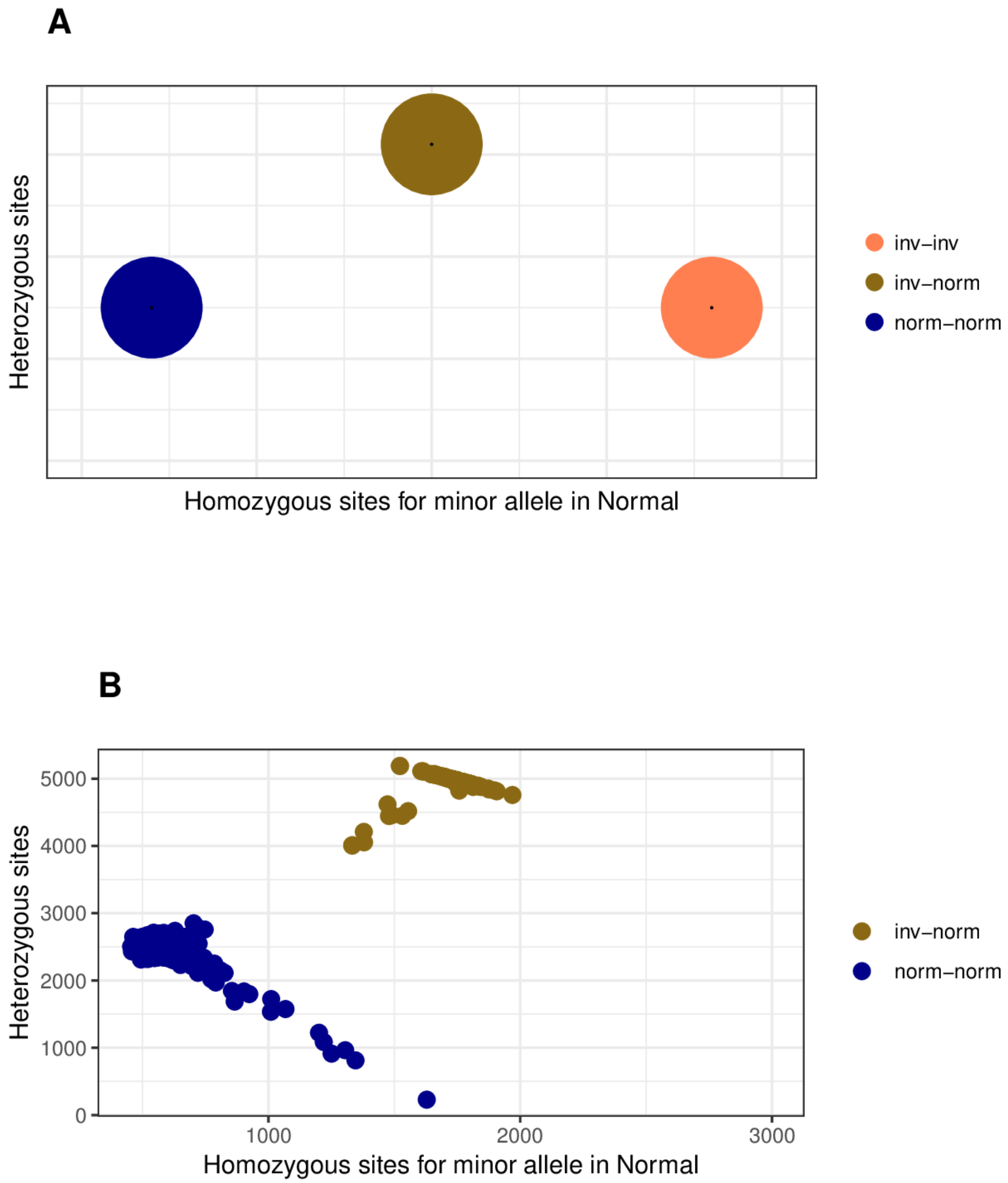

Figure 3: Cluster patterns, using all informative SNPs on Chromosome 1A, in each of the possible diploid karyotypes of a chromosome-wide inversion (i.e. norm-norm in dark blue, inv-norm in brown and inv-inv in orange, from left to right). The x-axis is the count trend of each karyotype for homozygous SNPs for the alternative allele in the normal phase. The y-axis is the count trend of each karyotype for heterozygous SNPs. Therefore, the expectations presented in the upper panel are based on the following assumptions: (i) inv-norm birds should have higher number of heterozygous SNPs across the chromosome 1A in comparison with inv-inv or norm-norm and (ii) inv-norm birds should have an intermediate number of homozygous SNPs for the minor allele in norm (i.e. "BB") in comparison with inv-inv or norm-norm. **A)** Expected clustering patterns. **B)** Cluster results from 2,296 great tits which were colored based on the classification from PCA analysis.

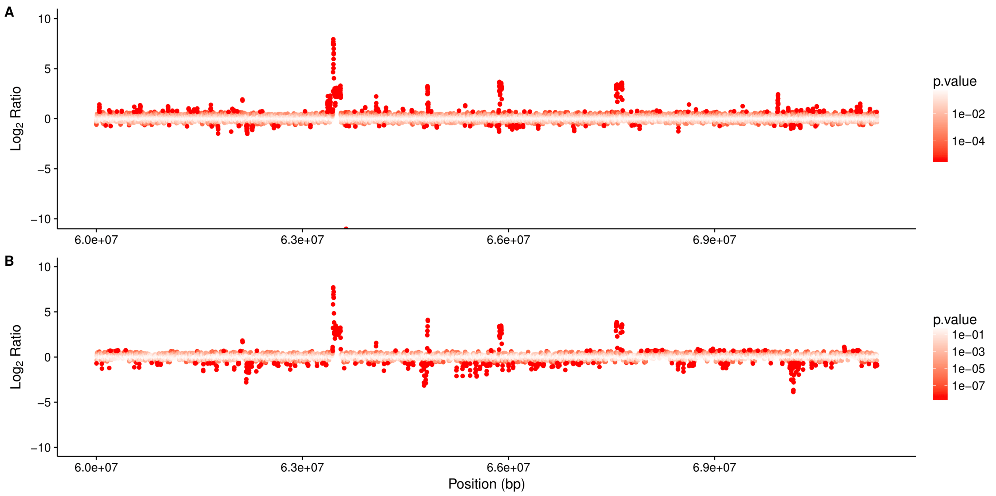

Figure 4: The x-axis represents the genomic coordinates of the CNV complex (i.e. downstream the inversion breakpoint) whereas the y-axis display the log_2 ratio that reflects the relative copy number across the complex (relative to a norm-norm bird). Thus, the anti-log of the log_2 ratio can be roughly interpreted as the absolute number of copies (i.e. if log_2 ratio = 3.333, then the anti-log is $2^{3.333} = \approx 10$ copies). A and B show respectively a female from France and a male from Belgium, which were classified as inv-norm based on sequencing data.

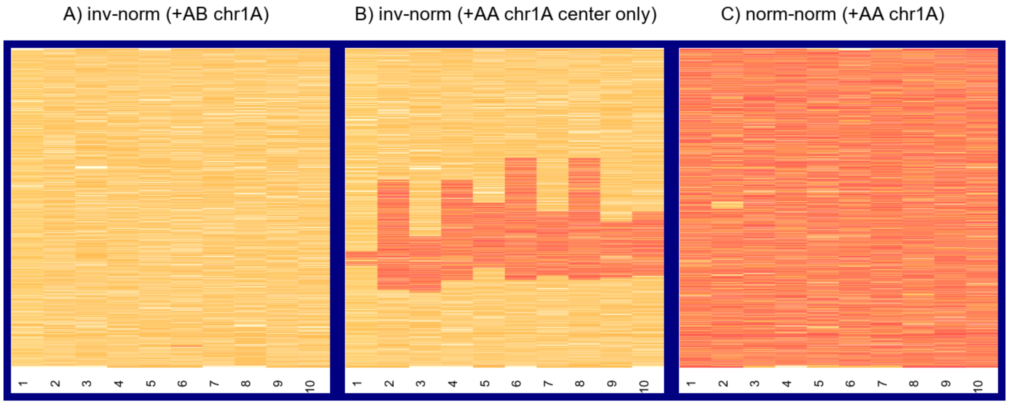

Figure 5: We used 4,124 informative SNPs (i.e. heterozygosity >0.6 in the inv-norm subpopulation), which are located in the center of the Chromosome 1A (20-60 Mb), to display the different inversion genotypes distributions in a heatmap. The SNP genotypes are represented by white ("BB"), light orange ("AB") and dark orange ("AA"), respectively. The distinct number of "AA" genotypes in the center of the inversion suggests different haplogroups in approximately 10% of the inv-norm birds (i.e. ten birds). **A)** Ten inv-norm birds selected randomly. **B)** Ten inv-norm birds displaying a distinct genotype distribution at the center of the inversion. **C)** Ten norm-norm birds selected randomly.

2 Supplementary methods

2.1 Classification confirmation for inversion carriers

Although PCA analysis is expected to produce clusters that distinguish inversion karyotypes due to genetic differentiation (i.e. both phases with the inversion, only one or absence of the inversion in both), we confirmed the inversion karyotypes using two sources of information. (i) Number of heterozygous SNPs and (ii) number of homologous SNPs for the minor allele in the normal phase, which are expected to form independent clusters for each inversion genotype in a scatter (XY) plot. For this confirmation strategy, we only used SNPs with heterozygosity value >0.6 in the subpopulation with higher values at eigenvector one (i.e. classified as inv-norm by PCA analysis). Therefore, we reclassified the birds as (i) norm-norm, (ii) inv-norm and (iii) inv-inv based on the XY plot for comparison with PCA classification.

2.2 Selection of the SNP used in the RFLP-PCR

All the SNPs supporting the inversion in the chromosome 1A were ranked by F_{ST} value. Thus, possible RFLP-PCR essays were simulated with the R/Bioconductor package DECIPHER [1]. The SNP AX-100689781 had the second highest F_{ST} value overall, but had the higher F_{ST} value among possible assays and was then carried forward for the subsequent primer design and enzyme search.

2.3 Primer design and enzyme search

In order to design a primer pair and pick a restriction enzyme which is able to differentiate genotypes at SNP AX-100689781, we first imported the reference sequence genome build 1.1 [2] with `readDNAStringSet` function from Biostrings R/Bioconductor package (v. 2.44.2) [3]. The sequence around the SNP was extracted and then written with `writeXStringSet` function, which is also available in Biostrings package. The candidate restriction enzyme was selected using the group-specific signatures pipeline available in the R/Bioconductor package DECIPHER manual [1]. The primers were designed using Primer3plus [4] and their quality was tested by NetPrimer (`http://www.premierbiosoft.com/netprimer`. The full nucleotide sequence of the amplicon (615 bp) can be copied directly from <NCBI>. The genotype-specific cutting patterns on the PCR amplicon (i.e. generated with the primers in Table 1) after digestion by the SspI enzyme is exemplified in the Sup Fig 4. The DNA of the selected animals was checked for quality and quantity with Qubit® Fluorometer.

Table 1: Primers used in the PCR-RFLP analysis.

	Sequence
Forward	GCCAGGCTCCTTAACATTTTG
Reverse	TCAGAGGGAACTGGATCTGC

Allele G digest

Enzymes: SspI

Length	5' Enzyme	5' Base	3' Enzyme	3' Base	Sequence
469	SspI	147	none	615	ATTTTTAAAA GAGTCATACC AAAGTGAAAA ATAAAAAGAA GGGAGTACAA AGGAAATTAC CCACCAACTG GTCTCCTTGT TCTAAGTGGG TCAGAACACG TCAGTATTTT CTAAATTTCT CCCACCTCCC AGCAGGAGCA GCATATTGAA GTGAAAATCA CAATTCAATG TTTATGGAGT ATCAATAACT CTAAAGAACT GCAGGTTGGC TGCATGGGGG TAAGAAAGAT GATTTCCCAC GTGCAGCAAC ACTTCACGGA TGGAAACAAT CTGCTCTTTC CTCTGTTGGT TATCCCTTGC CCTCCAAGTC CAACACACCA GTAGCAGCAC AGCCCTCACA GGTACAAAAA TGGCTTTCTT CTCATGGTTC CAGTATTTCT CCAGGCCATA CCAACCTGGA AAATTGTCCT CCTGAGCTCA TTCGGAGCCA CAGCAGTGGC TGTCCCCGAG CAGATCCAGT TCCCTCTGA
146	none	1	SspI	146	GCCAGGCTCC TTAACATTTT GAGGACAAAT TTGACTTCAA AGTTGTCATA GGCATGAAAA GGGACAAAAT AATTGTATTT ATTTTTATCA AGAAAGCCTC ATAGCTTGGC TTTCTGCTCA GACTAAAGCC AAGATGACAC CACAAT

Allele A digest

Enzymes: SspI

Length	5' Enzyme	5' Base	3' Enzyme	3' Base	Sequence
300	SspI	316	none	615	ATTTATGGAG TATCAATAAC TCTAAAGAAC TGCAGGTTGG CTGCATGGGG GTAAGAAAGA TGATTTCCCA CGTGCAGCAA CACTTCACGG ATGGAAACAA TCTGCTCTTT CCTCTGTTGG TTATCCCTTG CCCTCCAAGT CCAACACACC AGTAGCAGCA CAGCCCTCAC AGGTACAAAA ATGGCTTTCT TCTCATGGTT CCAGTATTTC TCCAGGCCAT ACCAACCTGG AAAATTGTCC TCCTGAGCTC ATTCGGAGCC ACAGCAGTGG CTGTCCCCGA GCAGATCCAG TTCCCTCTGA
169	SspI	147	SspI	315	ATTTTTAAAA GAGTCATACC AAAGTGAAAA ATAAAAAGAA GGGAGTACAA AGGAAATTAC CCACCAACTG GTCTCCTTGT TCTAAGTGGG TCAGAACACG TCAGTATTTT CTAAATTTCT CCCACCTCCC AGCAGGAGCA GCATATTGAA GTGAAAATCA CAATTCAAT
146	none	1	SspI	146	GCCAGGCTCC TTAACATTTT GAGGACAAAT TTGACTTCAA AGTTGTCATA GGCATGAAAA GGGACAAAAT AATTGTATTT ATTTTTATCA AGAAAGCCTC ATAGCTTGGC TTTCTGCTCA GACTAAAGCC AAGATGACAC CACAAT

Figure 6: Restriction enzyme digestion of the PCR amplicon considering a 2n state on the target region (diploid). As the region being analyzed mostly deviates from 2n, the real patterns may diverge in signal intensity as well. As the GG and AG genotypes represent mostly norm-norm and inv-norm respectively, norm-norm and inv-norm birds are expected to show two and four fragments respectively.

3 Supplementary results

3.1 Identification of the inversion carriers

We performed an additional test which relies on the assumption that informative SNPs should cluster birds with the same karyotype, based on the relative number of heterozygous SNPs and SNP genotypes homozygous for the minor allele in the normal phase (Sup Fig 2a). Thus, we classified the samples into (i) no inversion as norm-norm (ii) one inverted phase as inv-norm and (iii) two inverted phases as inv-inv (not found in this population) as in the PCA test. The test reflected the PCA clustering results and we therefore classified 117 birds as inv-norm and 2,179 as norm-norm (Sup Fig 2b).

3.2 Quality of the SNPs used in the LD analysis

To make sure that the high incidence of "AA" genotypes in the center of the inversion for some inv-norm birds is not due to low quality markers, we compared the consistency of genotypes in the reference genome animal which was genotyped twice. We split chromosome 1A into 500 tiles (≈140kb each) and estimated the percentage of concordant genotypes in both assays for each tile. We could not find any indication of low quality SNPs within the R^2 LD block (i.e no lower genotyping quality in the center of the chromosome, Sup Fig 4, t-test p-value = 0.84).

3.3 Genes overlapping the CNVR at the CNV complex

The SNP within the CNV complex, used for inversion detection by PCR-RFLP (high F_{ST} value within the inversion), is placed at the first intron of the *PIK3C2G* gene which has crucial role on signaling pathways [5]. Nevertheless, the CNV complex in the inversion breakpoint is a gene-rich genomic interval that encompasses 32 genes (16 with known gene names) that are related to a wide range of processes (Table 1). These genes or its paralogs translate proteins involved in the cell cycle (*PDE3A*, *RERG* and *PIK3C2G*) [6, 7, 5], protein trafficking (*PIK3C2G*) [5], muscle contraction (*CALD1*) [8], recurrent translocation in cancer (*LMO3*) [9], spliceosome activity (*STRAP*) [10, 11], brain development (*PLEKHA5*) [12], glucose metabolism (*IAPP*) [13], oxygen sensing in blood cells (*BPGM*) [14], fat production (*MGST1*) [15], signalling (*EPS8* and *RERGL*) [16, 17], solute transport (*SLC15A5*) [18], synapse formation and apoptosis (*PTPRO*) [19, 20], energy metabolism (*DERA*), [21] and even pigmentation by affecting Polycomb activity (*AEBP2*) [22, 23], which is a key process in gene silencing [24].

To make sure the higher rate of informative SNPs at the CNV complex is not driven by low quality genotypes at this region, we compared the percentage of consistent genotypes at the complex with the genotypes in other regions of the chromosome 1A. We found no significant difference (t-test, p-value = 0.75), what suggests that the number of false positives in this region is not higher than other regions in the chromosome 1A.

Table 2: Genes overlapping the CNV complex at the downstream breakpoint of the inversion.

Chromosome	Start	End	Width	Name
chr1A	64843171	64844337	1167	LOC107204104
chr1A	64861670	64908113	46444	LOC107205143
chr1A	64874841	64878856	4016	IAPP
chr1A	64919923	64938780	18858	LOC107205182
chr1A	64947738	64989258	41521	LOC107204204
chr1A	64999708	65223576	223869	PDE3A
chr1A	65224970	65233165	8196	LOC107205022
chr1A	65236702	65339065	102364	LOC107205021
chr1A	65274559	65279283	4725	LOC107205023
chr1A	65355652	65396498	40847	LOC107204113
chr1A	65516912	65560642	43731	AEBP2
chr1A	65577008	65743662	166655	PLEKHA5
chr1A	65862206	66091155	228950	PIK3C2G
chr1A	66109620	66118841	9222	RERGL
chr1A	66427883	66437729	9847	LOC107204286
chr1A	66557323	66617748	60426	LMO3
chr1A	66647333	66649964	2632	LOC107204290
chr1A	66674727	66682085	7359	MGST1
chr1A	66709327	66739543	30217	SLC15A5
chr1A	66789556	66833259	43704	DERA
chr1A	66836525	66844259	7735	STRAP
chr1A	66845766	66857357	11592	LOC107204111
chr1A	66873268	67003015	129748	EPS8
chr1A	67004993	67150264	145272	PTPRO
chr1A	67023437	67032017	8581	LOC107204503
chr1A	67191246	67291500	100255	RERG
chr1A	67330974	67366580	35607	LOC107204153
chr1A	67377799	67401512	23714	LOC107204567
chr1A	67400647	67409947	9301	LOC107204566
chr1A	67410594	67581825	171232	CALD1
chr1A	67622020	67640854	18835	LOC107204149
chr1A	67646418	67680793	34376	BPGM

3.4 Patterns in split reads supporting the CNV complex

We manually checked the reads overlapping CNVs which are located nearby to the downstream breakpoint of the inversion (Sup Table 3). Interestingly, we found read pairs at the breakpoints of the CNVs 1, 2 and 3 to support their structural rearrangement into a CNV complex (Sup Figure 5). However, although the inversion breakpoint is relatively clear in the SNP-array based results (Figure 1), CNVs identified with sequencing data indicate that the inversion breakpoint may be placed at the CNV complex. These CNVs belonging to the CNV complex are nearby to gaps in the reference genome, which adds another layer of complexity to the interpretation of these variants. Moreover, it is not completely clear how the ≈ 10 copies of the complex are distributed across the genome (e.g. *in tandem* or not). Thus, the actual boundaries of the inversion might differ from the breakpoints found in SNP array results.

Table 3: Sequencing coverage in two inv-norm birds

CNV id	CNV location	PHRED quality	French coverage	Belgium coverage
CNV1	65.87-65.90	8677.93	112.832	86.658
CNV2	67.56-67.58	8352.07	110.254	102.649
CNV3	67.64-67.65	8677.93	113.469	103.582
CNVup1	63.44-63.46	9274.26	2105.23	2074.36
CNVup2	63.46-63.56	6293.79	83.6796	68.7332

French coverage = read depth of the sequenced sample from a French population (id = 233, 1A average coverage = 13.15); Belgium coverage = read depth of the sequenced sample from a Belgium population (id = 973, 1A average coverage = 9.55)

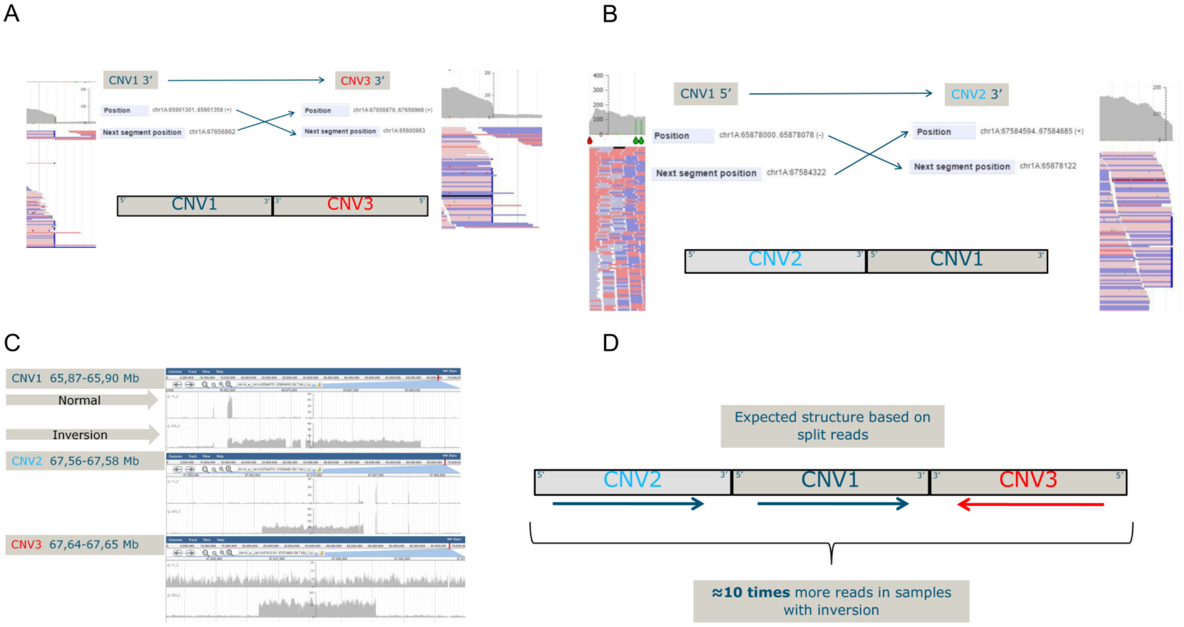

Figure 7: **A)** Split reads supporting the structural rearrangement between CNV1 (65.87-65.90) and CNV3 (67.64-67.65) **B)** Split reads supporting the structural rearrangement between CNV2 (67.56-67.58) and CNV1 (65.87-65.90).

References

[1] E.S. Wright. Using decipher v2.0 to analyze big biological sequence data in r. *The R Journal*, 8, 2016.

[2] Veronika N. Laine, Toni I. Gossmann, Kyle M. Schachtschneider, Colin J. Garroway, Ole Madsen, Koen J. F. Verhoeven, Victor de Jager, Hendrik-Jan Megens, Wesley C. Warren, Patrick Minx, Richard P. M. A. Crooijmans, Pádraic Corcoran, Frank Adriaensen, Eduardo Belda, Andrey Bushuev, Mariusz Cichon, Anne Charmantier, Niels Dingemanse, Blandine Doligez, Tapio Eeva, Kjell Einar Erikstad, Slava Fedorov, Michaela Hau, Sabine Hille, Camilla Hinde, Bart Kempenaers, Anvar Kerimov, Milos Krist, Raivo Mand, Erik Matthysen, Reudi Nager, Claudia Norte, Markku Orell, Heinz Richner, Tore Slagsvold, Vallo Tilgar, Joost Tinbergen, Janos Torok, Barbara Tschirren, Tera Yuta, Ben C. Sheldon, Jon Slate, Kai Zeng, Kees van Oers, Marcel E. Visser, and Martien A. M. Groenen. Evolutionary signals of selection on cognition from the great tit genome and methylome. *Nat. Commun.*, 7:10474, jan 2016.

[3] H. Pagès, P. Aboyoun, R. Gentleman, and S. DebRoy. Biostrings: String objects representing biological sequences, and matching algorithms., 2017.

[4] Andreas Untergasser, Harm Nijveen, Xiangyu Rao, Ton Bisseling, René Geurts, and Jack A M Leunissen. Primer3Plus, an enhanced web interface to Primer3. *Nucleic Acids Res.*, 35(Web Server issue):W71–4, jul 2007.

[5] Magdalena Rozycka, Yong-Jie Lu, Richard A. Brown, Mike R. Lau, Janet M. Shipley, and Michael J. Fry. cDNA Cloning of a Third Human C2-Domain-Containing Class II Phosphoinositide 3-Kinase, PI3K-C2γ, and Chromosomal Assignment of This Gene (PIK3C2G) to 12p12. *Genomics*, 54(3):569–574, dec 1998.

[6] Najma Begum, Weixing Shen, and Vincent Manganiello. Role of PDE3A in regulation of cell cycle progression in mouse vascular smooth muscle cells and oocytes: implications in cardiovascular diseases and infertility. *Curr. Opin. Pharmacol.*, 11(6):725–729, dec 2011.

[7] Weilin Zhao, Ning Ma, Shumin Wang, Yingxi Mo, Zhe Zhang, Guangwu Huang, Kaoru Midorikawa, Yusuke Hiraku, Shinji Oikawa, Mariko Murata, and Kazuhiko Takeuchi. RERG suppresses cell proliferation, migration and angiogenesis through ERK/NF-κB signaling pathway in nasopharyngeal carcinoma. *J. Exp. Clin. Cancer Res.*, 36(1):88, dec 2017.

[8] M P Walsh. Calmodulin and the regulation of smooth muscle contraction. *Mol. Cell. Biochem.*, 135(1):21–41, jun 1994.

[9] Jennifer Chambers and Terence H. Rabbitts. LMO2 at 25 years: a paradigm of chromosomal translocation proteins. *Open Biol.*, 5(6):150062, jun 2015.

[10] Hyun-A. Seong, Haiyoung Jung, Hueng-Sik Choi, Kyong-Tai Kim, and Hyunjung Ha. Regulation of Transforming Growth Factor-β Signaling and PDK1 Kinase Activity by Physical Interaction between PDK1 and Serine-Threonine Kinase Receptor-associated Protein. *J. Biol. Chem.*, 280(52):42897–42908, dec 2005.

[11] Ashwin Chari, Monika M. Golas, Michael Klingenhäger, Nils Neuenkirchen, Bjoern Sander, Clemens Englbrecht, Albert Sickmann, Holger Stark, and Utz Fischer. An Assembly Chaperone Collaborates with the SMN Complex to Generate Spliceosomal SnRNPs. *Cell*, 135(3):497–509, oct 2008.

[12] Kenichiro Yamada, Noriko Nomura, Arisa Yamano, Yasukazu Yamada, and Nobuaki Wakamatsu. Identification and characterization of splicing variants of PLEKHA5 (Plekha5) during brain development. *Gene*, 492(1):270–275, jan 2012.

[13] H Mulder, B Ahrén, and F Sundler. Islet amyloid polypeptide and insulin gene expression are regulated in parallel by glucose in vivo in rats. *Am. J. Physiol.*, 271(6 Pt 1):E1008–14, dec 1996.

[14] N. Petousi, R. R. Copley, T. R. J. Lappin, S. E. Haggan, C. M. Bento, H. Cario, M. J. Percy, P. J. Ratcliffe, P. A. Robbins, M. F. McMullin, P. Donnelly, J. Bell, D. Bentley, G. McVean, P. Ratcliffe, J. Taylor, A. Wilkie, P. Donelly, J. Broxholme, D. Buck, J.-B. Cazier, R. Cornall, L. Gregory, J. Knight, G. Lunter, G. McVean, I. Tomlinson, A. Wilkie, D. Buck, C. Allan, M. Attar, A. Green, L. Gregory, S. Humphray, Z. Kingsbury, S. Lamble, L. Lonie, A. Pagnamenta, P. Piazza, G. Polanco, A. Trebes, G. McVean, P. Donnelly, J.-B. Cazier, J. Broxholme, R. Copley, S. Fiddy, R. Grocock, E. Hatton, C. Holmes, L. Hughes, P. Humburg, A. Kanapin, S. Lise, G. Lunter, H. Martin, L. Murray, D. McCarthy, A. Rimmer, N. Sahgal, B. Wright, and C. Yau. Erythrocytosis associated with a novel missense mutation in the BPGM gene. *Haematologica*, 99(10):e201–e204, oct 2014.

[15] Mathew D. Littlejohn, Kathryn Tiplady, Tania A. Fink, Klaus Lehnert, Thomas Lopdell, Thomas Johnson, Christine Couldrey, Mike Keehan, Richard G. Sherlock, Chad Harland, Andrew Scott, Russell G. Snell, Stephen R. Davis, and Richard J. Spelman. Sequence-based Association Analysis Reveals an MGST1 eQTL with Pleiotropic Effects on Bovine Milk Composition. *Sci. Rep.*, 6(1):25376, jul 2016.

[16] Letizia Lanzetti, Vladimir Rybin, Maria Grazia Malabarba, Savvas Christoforidis, Giorgio Scita, Marino Zerial, and Pier Paolo Di Fiore. The Eps8 protein coordinates EGF receptor signalling through Rac and trafficking through Rab5. *Nature*, 408(6810):374–377, nov 2000.

[17] J. Colicelli. Human RAS Superfamily Proteins and Related GTPases. *Sci. Signal.*, 2004(250):re13–re13, sep 2004.

[18] P. J. Hoglund, K. J. V. Nordstrom, H. B. Schioth, and R. Fredriksson. The Solute Carrier Families Have a Remarkably Long Evolutionary History with the Majority of the Human Families Present before Divergence of Bilaterian Species. *Mol. Biol. Evol.*, 28(4):1531–1541, apr 2011.

[19] Wei Jiang, Mengping Wei, Mengna Liu, Yunlong Pan, Dong Cao, Xiaofei Yang, and Chen Zhang. Identification of Protein Tyrosine Phosphatase Receptor Type O (PTPRO) as a Synaptic Adhesion Molecule that Promotes Synapse Formation. *J. Neurosci.*, 37(41):9828–9843, oct 2017.

[20] Caihong Liang, Xiaochen Wang, Jianping Hu, Xiaoqing Lian, Tiantian Zhu, Hui Zhang, Ning Gu, and Jun Li. PTPRO Promotes Oxidized Low-Density Lipoprotein Induced Oxidative Stress and Cell Apoptosis through Toll-Like Receptor 4/Nuclear Factor κB Pathway. *Cell. Physiol. Biochem.*, 42(2):495–505, 2017.

[21] Lisa Salleron, Giovanni Magistrelli, Camille Mary, Nicolas Fischer, Amos Bairoch, and Lydie Lane. DERA is the human deoxyribose phosphate aldolase and is involved in stress response. *Biochim. Biophys. Acta - Mol. Cell Res.*, 1843(12):2913–2925, dec 2014.

[22] Anne Grijzenhout, Jonathan Godwin, Haruhiko Koseki, Michal Ryszard Gdula, Dorota Szumska, Joanna F. McGouran, Shoumo Bhattacharya, Benedikt M. Kessler, Neil Brockdorff, and Sarah Cooper. Functional analysis of AEBP2, a PRC2 Polycomb protein, reveals a Trithorax phenotype in embryonic development and in ESCs. *Development*, 143(15):2716–2723, aug 2016.

[23] Hana Kim, Keunsoo Kang, Muhammad B. Ekram, Tae-Young Roh, and Joomyeong Kim. Aebp2 as an Epigenetic Regulator for Neural Crest Cells. *PLoS One*, 6(9):e25174, sep 2011.

[24] Shahram Golbabapour, Nazia Abdul Majid, Pouya Hassandarvish, Maryam Hajrezaie, Mahmood Ameen Abdulla, and A. Hamid A. Hadi. Gene Silencing and Polycomb Group Proteins: An Overview of their Structure, Mechanisms and Phylogenetics. *Omi. A J. Integr. Biol.*, 17(6):283–296, jun 2013.
